# Diffusion weighted magnetic resonance spectroscopy revealed neuronal specific microstructural alterations in Alzheimer’s disease

**DOI:** 10.1093/braincomms/fcae026

**Published:** 2024-02-01

**Authors:** Nicola Spotorno, Chloé Najac, Olof Strandberg, Erik Stomrud, Danielle van Westen, Markus Nilsson, Itamar Ronen, Oskar Hansson

**Affiliations:** Clinical Memory Research Unit, Department of Clinical Sciences, Lund University, Malmö, Lund 22184, Sweden; Department of Radiology, C.J. Gorter MRI Center, Leiden University Medical Center, Leiden 2333, The Netherlands; Clinical Memory Research Unit, Department of Clinical Sciences, Lund University, Malmö, Lund 22184, Sweden; Clinical Memory Research Unit, Department of Clinical Sciences, Lund University, Malmö, Lund 22184, Sweden; Memory Clinic, Skåne University Hospital, Malmö 20502, Sweden; Image and Function, Skane University Hospital, Lund 22185, Sweden; Diagnostic Radiology, Institution for Clinical Sciences, Lund University, Lund 22185, Sweden; Diagnostic Radiology, Institution for Clinical Sciences, Lund University, Lund 22185, Sweden; Clinical Imaging Sciences Centre, Brighton and Sussex Medical School, Falmer BN1 9RR, UK; Clinical Memory Research Unit, Department of Clinical Sciences, Lund University, Malmö, Lund 22184, Sweden; Memory Clinic, Skåne University Hospital, Malmö 20502, Sweden

**Keywords:** diffusion-weighted magnetic resonance spectroscopy, tau, *N*-acetyl-aspartate, astrocytes, Alzheimer’s disease

## Abstract

In Alzheimer’s disease, reconfiguration and deterioration of tissue microstructure occur before substantial degeneration become evident. We explored the diffusion properties of both water, a ubiquitous marker measured by diffusion MRI, and *N*-acetyl-aspartate, a neuronal metabolite probed by diffusion-weighted magnetic resonance spectroscopy, for investigating cortical microstructural changes downstream of Alzheimer’s disease pathology. To this aim, 50 participants from the Swedish BioFINDER-2 study were scanned on both 7 and 3 T MRI systems. We found that in cognitively impaired participants with evidence of both abnormal amyloid-beta (CSF amyloid-beta42/40) and tau accumulation (tau-PET), the *N*-acetyl-aspartate diffusion rate was significantly lower than in cognitively unimpaired participants (*P* < 0.05). This supports the hypothesis that intraneuronal tau accumulation hinders diffusion in the neuronal cytosol. Conversely, water diffusivity was higher in cognitively impaired participants (*P* < 0.001) and was positively associated with the concentration of myo-inositol, a preferentially astrocytic metabolite (*P* < 0.001), suggesting that water diffusion is sensitive to alterations in the extracellular space and in glia. In conclusion, measuring the diffusion properties of both water and *N*-acetyl-aspartate provides rich information on the cortical microstructure in Alzheimer’s disease, and can be used to develop new sensitive and specific markers to microstructural changes occurring during the disease course.

## Teaser

The authors showed that measuring the diffusion properties of both water and *N*-acetyl-aspartate, using diffusion-weighted MRI and diffusion-weighted spectroscopy, respectively, provides rich information on both intraneuronal and extracellular microstructural properties of cortical structures in Alzheimer’s disease.

## Graphical Abstract

**Graphical Abstract:**
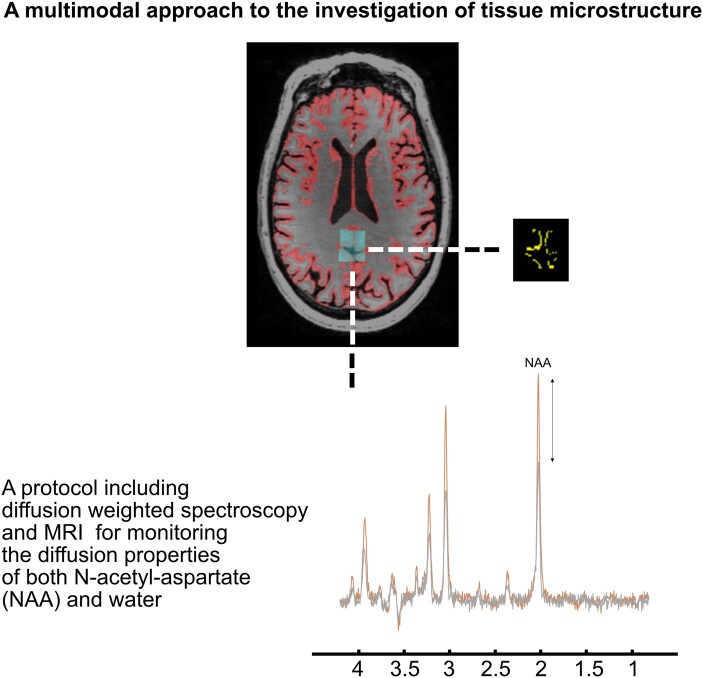

## Introduction

Over the course of the pathological cascade of Alzheimer’s disease (AD), misfolding and accumulation of both amyloid-beta (Aβ) and tau lead to profound changes in cortical microstructure.^1^ Such changes are, however, difficult to detect using conventional imaging prior to overt atrophy, measurable with morphological metrics from structural MRI.

In recent years, several works applied diffusion MRI (DW-MRI) for investigating cortical microstructure in Alzheimer’s disease.^2-4^ DW-MRI probes the displacement of water molecules, which are present in both intra- and extracellular compartments and therefore cannot reliably identify the compartment(s) affected by a pathological process. For example, in AD, hyperphosphorylated tau accumulation in neurons and glial activation in response to neuroinflammation result in significant changes in the cytomorphology of neurons and glia (microglia and astrocytes), respectively.^5,6^ These changes are expected to affect the diffusion rate of water inside neurons and glia, and it is not known whether the separate contributions of neurons and glia cells can be disentangled in DW-MRI experiments. Modelling frameworks proposed to overcome some limitations of DW-MRI, providing more compartment-specific information.^7,8^ Such approaches, however, are heavily based on generalized assumptions regarding cytomorphological features that do not always hold, especially in pathological conditions.^9^ In contrast, diffusion-weighted MR spectroscopy (DW-MRS) relies on the specificity of MR spectroscopy (MRS) to the chemical composition of different brain metabolites. This enables reliable quantification of the diffusion properties of MR-measurable metabolites. These are predominantly intracellular and preferentially localized in specific brain cell populations. *N*-acetyl-aspartate (NAA) is a metabolite predominantly found in neurons in the central nervous system.^10^

Previous DW-MRS studies in neurological disorders such as multiple sclerosis^11,12^ and amyotrophic lateral sclerosis^13^ harnessed the specificity of NAA to neurons to track the neurodegenerative process by specifically showing differences in the diffusion of NAA and suggesting the diffusion rate of NAA and water can be differentially affected by different pathological phenomena such as neurodegeneration and neuroinflammation.^11,12^

In this work, we tested the sensitivity of DW-MRS to Alzheimer’s disease pathology by investigating the diffusion of NAA in the posterior cingulate cortex of Aβ-negative cognitively unimpaired individuals as well as in cognitively impaired patients with evidence of significant Aβ and tau accumulation. We also compared the diffusion rate of NAA with the diffusion rate of water as well as with the concentration of commonly quantified metabolites in neurodegenerative diseases, such as choline compounds (tCho), associated with cell membrane breakdown,^14,15^ myo-inositol (mIns), a metabolite almost exclusively localized in astrocytes^16,17^ and NAA itself. We hypothesized that in the Alzheimer’s disease pathological cascade, the presence of tau aggregates hinders the diffusion of NAA in the neuronal cytosol, resulting in a reduced apparent diffusion coefficient (ADC_NAA_). In addition, we anticipated that the rate of water diffusion would correlate with markers of both neurodegeneration and inflammation, namely the concentrations of NAA and of glial metabolites.

## Materials and methods

### Participants

Twenty-five cognitively unimpaired participants (CU) and 25 cognitive impaired participants (CI) from the Swedish BioFINDER-2 study (NCT03174938)14 were included. The cognitively unimpaired individuals did not have evidence of Aβ pathology according to a previously reported CSF Aβ42/40 cut-off14, while cognitively impaired individual had evidence of both Aβ and tau pathology according to previously reported CSF Aβ42/40 and tau-PET cut-off14,15, respectively (see Supplementary material for the full inclusion and exclusion criteria). Twelve participants were excluded from the study due to imaging artefacts or excessive motion during scan. Therefore, 38 participants (19 CU and 19 CI) were included in the final cohort. Three participants (one cognitively unimpaired and two cognitively impaired) were not included in the analysis involving DW-MRI data because the data were not available, leaving a total of 35 participants for these analyses: 18 CU and 17 CI. The acquisitions were performed between 2019 and 2021. Demographic and clinical characteristics of the final cohort are summarized in Table 1. All subjects gave written informed consent according to the Declaration of Helsinki, and the study was approved by the Ethical Review Board of Lund, Sweden.

**Table 1:** Demographic summary of the study cohort

	CU	CI
*n* (% of female)^a^	19 (53%)	19 (47%)
Age	67 (7)	68 (8)
Years of education	12 (4)	14 (5)
MMSE	29 (1)	22 (4)^b^
Tau-PET uptake in the volume of interest	0.9 (0.1)	2.1 (0.5)^b^
Grey matter in the volume of interest	61% (4%)	49% (6%)^b^
White matter in the volume of interest	32% (4%)	30% (6%)
CSF in the volume of interest	7% (3%)	21% (9%)^b^

Values are given as mean (standard deviation) except for the number of participants (percentage of female).

MMSE, Mini Mental State Examination; CU, cognitively unimpaired participants; CI, cognitively impaired participants; volume of interest, MRS volume positioned in the precuneus–posterior cingulate cortex.

^a^Water diffusion MRI data were not available for three participants (one CU and two CI). Therefore, the analyses including water diffusion MRI data are based on 35 participants (18 CU and 17 CI).

^b^Statistically significant difference between CU and CI (*P* < 0.05).

## Imaging protocol and analysis

### MRS–MRI 7 T protocol

7 T MRI scans were performed on a Philips Achieva whole-body scanner (Philips Healthcare, Best, The Netherlands). The scan protocol consisted of a short survey scan and a sensitivity encoding (SENSE) reference scan followed by a 3D T_1_-weighted (T_1_w) scan [magnetization-prepared rapid gradient-echo sequence (MPRAGE), inversion time (TI) = 898 ms; flip angle = 7°; TR/TE = 5.0/2.2 ms; resolution = 1 × 1 × 1 mm^3^, field of view = 246 × 174 × 246 mm^3^ in the anterior–posterior (AP), right–left (RL), and foot-head directions (FH), SENSE factor = 2 (AP) and 2.5 (RL)].

‘Single-volume water suppressed MRS data’ were acquired with sLASER [TR/TE = 4000/26 ms, number of time domain points (np) = 1024, spectral bandwidth (SW) = 3000 Hz, number of scan averages (NSA) = 48]. A 20 × 20 × 20 mm^3^ volume of interest (VOI) was positioned in the precuneus–posterior cingulate cortex region (PCC, Supplementary Fig. 1) using the T_1_w images. The macromolecule baseline employed in the study has been previously acquired using a 7 T scanner^18^ and it is publicly available at MRSHub (https://github.com/mrshub/mm-consensus-data-collection; see Supplementary methods for additional details).

‘Single-volume water and metabolite DW-MRS data’ were acquired using a DW-sLASER sequence^19^ (TE = 101 ms, np = 1024, sw = 3000 Hz). DW gradients were applied along three orthogonal directions using two *b*-values. True *b*-values were estimated using sequence chronograms to account for cross-terms between localization and spoiler gradients and DW gradients (*b* = 639 and 4433 s/mm^2^). To minimize signal fluctuations due to cardiac pulsation, cardiac triggering was performed using a peripheral pulse unit (trigger delay: 200 ms, TR: 4 cardiac cycles). See Supplementary material for additional details.

### MRI 3 T protocol

Scans were performed on a 3 T MRI MAGNETOM Prisma scanner (Siemens Healthcare, Erlangen, Germany), equipped with a 64-channel head coil.

DW-MRI scans: A diffusion-weighted single-shot echo-planar imaging sequence was used and 104 diffusion-weighted imaging volumes were acquired with the following parameters: TR/TE = 3500/73 ms, resolution = 2 × 2 × 2 mm^3^, field of view (AP/RL/FH) = 220 × 220 × 124 mm^3^, *b* = 0, 100, 1000 and 2500 s/mm^2^ distributed over 2, 6, 32 and 64 directions, respectively, 2-fold parallel acceleration and partial Fourier factor = 7/8. A T_1_w MPRAGE image was also acquired (flip angle: 9°; TR/TE = 1900/2.54 ms; field of view = 256 × 256 × 176 mm^3^; resolution = 1 × 1 × 1 mm^3^; 2-fold parallel acceleration). For three participants, the DW-MRI data were not available.

### PET protocol

Tau-PET was performed on Discovery MI scanner (GE healthcare) using the radio ligand ^18^F-RO948. Images were acquired 70 to 90 minutes after injection of 370 MBq ^18^F-RO948. Pre-processing and generation of standardized uptake value ratios (SUVR) maps were carried out as previously described using the inferior cerebellar grey matter as reference region.^20,21^ For all subjects, tau positivity was defined based on tau-PET uptake from a temporal composite region using a previously published cut-off of 1.36.^20^

## MRS/MRI data processing and analysis

### 7 T data analysis

#### Structural data

T_1_w images were segmented into GM, WM and cerebrospinal fluid (CSF) maps using FSL (Brain extraction Tool^22^ and FAST^23^ algorithm in the FMRIB Software Library). Each voxel contained a value in the range between 0 and 1 that represented the fraction of each tissue type. A threshold of 0.5 was used to generate GM, WM and CSF masks. An in-house Matlab routine (MathWorks, Inc., MA, USA) was subsequently used to quantify the tissue volumes within each spectroscopic VOI and generate VOI-only tissue masks.

#### MRS data

MRS data were fitted using LCModel^24^ using a basis-set generated with FID-A.^25^ Ratios to total creatine (tCr = creatine (Cr) + phosphocreatine (PCr)) were estimated.

#### DW-MRS data

Individual spectra were corrected for eddy currents and phase/frequency drifts using in-house Matlab routines as previously described.^26^ DW spectra were quantified with LCModel,^24^ resulting in signal amplitude of *N*-acetyl-aspartate (NAA), for each spectrum. *N*-acetyl-aspartate ADC (ADC_NAA_) values were finally calculated according to standard procedure.

### 3 T data analysis

#### Structural data

MPRAGE images were preprocessed using FreeSurfer pipeline (version 6.0, https://surfer.nmr.mgh.harvard.edu) that included correction for intensity homogeneity,^27^ brain extraction^28^ and tissue segmentation.^29^

#### DW-MRI data

DW-MRI data were processed using a combination of open-source algorithms. The acquired images were corrected for susceptibility-induced distortion using images acquired with opposite phase encoding gradient polarities. Motion and eddy currents were corrected using FSL tools (FMRIB Software Library, version 6.0.4; Oxford, UK). The diffusion tensor model was fitted and voxel-wise water apparent diffusion coefficient (ADC_water_) maps were computed using MRtrix3^30^ routines.

#### Surface projection of ADC_water_

The diffusion images were co-registered using the averaged *b* = 0 volumes to each subject’s MPRAGE volume using ANTs tools (v2.1). The ADC_water_ maps were warped to the same space and a surface projection of ADC_water_ was generated using FreeSurfer commands as previously reported.^3^

### Cross-platform data co-registration

Each subject’s 3 T MPRAGE and SUVR maps were rigidly aligned to the corresponding 7 T MPRAGE map using ANTs. The surface representation of ADC_water_ was projected in volumetric space and warped to the 7 T image. Subsequently, the MRS volume mask was superimposed onto the ADC_water_ and SUVR maps, and the median ADC_water_ and SUVR values were extracted from the same location.

### Statistical analysis

The relationships between demographic variables and clinical status were evaluated with chi-square and Student’s *t*-test (Table 1). Multiple linear regression models were employed to investigate the differences in both ADC_NAA_ and relative concentration of metabolites as well as in ADC_water_ between the CI and CU participants. Multiple regression models were also employ to investigate the association between ADC_(NAA or water)_ and the relative concentration of metabolites. Age and sex were included as covariates. When using an (DW-)MRS-derived metric as the dependent variable, the percentage of WM in the MRS voxel (WM_frac_) was included as a covariate to account for the potential confounding effect of a variable proportion of GM and WM in the volume of interest (e.g. ADC_NAA_ ∼ Group + Age + Sex + WM_frac_).

## Results

### Both ADC_NAA_ and ADC_water_ differed between diagnostic groups and are negatively associated with one another

**Figure 1:**
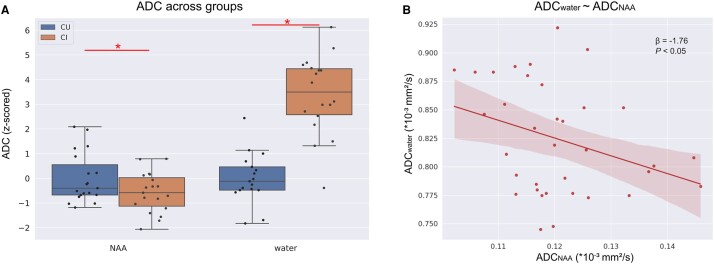
**Apparent diffusion coefficient of NAA and water across groups**. ADC, apparent diffusion coefficient; NAA, *N*-acetyl-aspartate; CU, cognitively unimpaired participants; CI, cognitively impaired participants; *statistically significant difference at *P* < 0.05 as tested with multiple linear regressions. (**A**) ADC of both NAA and water across groups (ADC_NAA_: *β* = −0.007, *P* < 0.05; ADC_water_: *β* = 0.080, *P* < 0.001). Both ADCs were computed from the same location (precuneus–posterior cingulate cortex). For representation purposes, the ADC has been *Z*-scored. (**B**) Association between the ADC of NAA (ADC_NAA_) and the ADC of water (ADC_water_) extracted from the same location (precuneus–posterior cingulate cortex). *β*- and *P*-values were derived from a multiple linear regression.

ADC_NAA_ was significantly lower in the CI group compared with the CU group. In contrast, ADC_water_ was significantly higher in the CI group (ADC_NAA_: *β* = −0.007, *P* < 0.05; ADC_water_: *β* = 0.080, *P* < 0.001, see Fig. 1A). This suggested that the group differences in ADC_NAA_ and in ADC_water_ reflect different biological phenomena. Furthermore, ADC_NAA_ and ADC_water_ showed a significant negative correlation (*β* = −1.971, *P* < 0.05; see Fig. 1B and Supplementary Table 1).

### ADC_water_ but not ADC_NAA_ is associated with the relative concentration of myo-inositol in the same region

**Figure 2:**
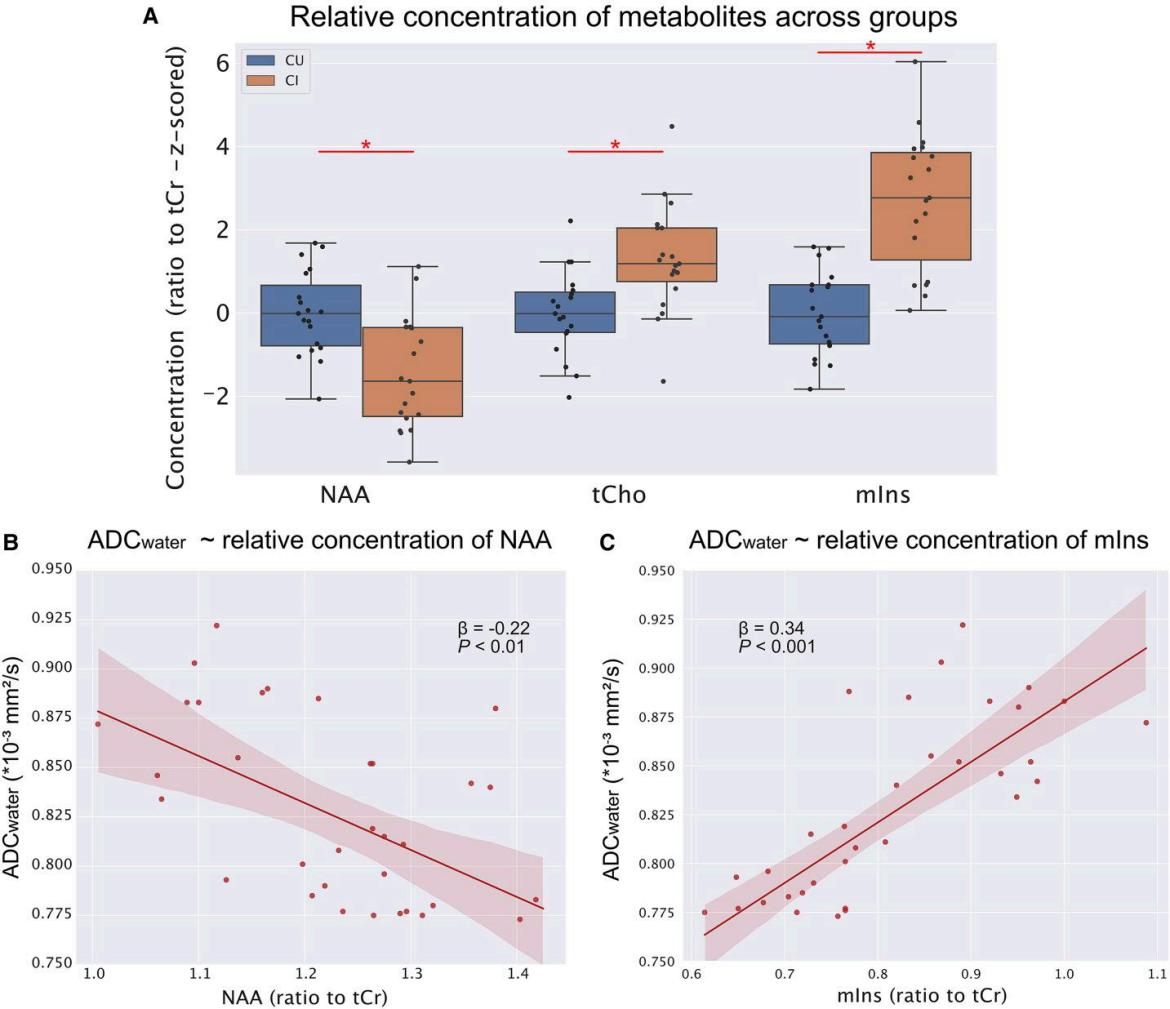
**Relative concentration of metabolites and their association with the ADC of water**. ADC, apparent diffusion coefficient; NAA, *N*-acetyl-aspartate; tCr, total creatine; tCho, total choline; mIns, myo-inositol; CU, cognitively unimpaired participants; CI, cognitively impaired participants; *statistically significant difference at *P* < 0.05 as tested with multiple linear regressions. The concentration of metabolites is expressed as ratio to tCr. (**A**) Difference in the relative concentration of commonly quantified metabolites across groups (NAA: *β* = −0.13, *P* < 0.001; tCho: *β* = 0.03, *P* < 0.01; mIns: *β* = 0.17, *P* < 0.001) (*Z*-scored for representation purposes). (**B**) Negative association between the relative concentration of NAA and the cortical ADC of water (ADC_water_) extracted from the same location of the MRS-VOI. (**C**) Positive association between the relative concentration of mIns and the cortical ADC_water_ extracted from the same location of the MRS voxel. *β*- and *P*-values were derived from a multiple linear regression.

To further investigate the biological underpinning of the pathology-related changes in ADC_NAA_ and ADC_water_, we examined the relative concentration of metabolites from MRS and their associations with both ADC_NAA_ and ADC_water_. Compared with the CU group, the relative concentration of both myo-inositol and total choline was higher in the CI group and the concentration of NAA was lower (NAA: *β* = −0.13, *P* < 0.001; tCho: *β* = 0.03, *P* < 0.01; mIns: *β* = 0.17, *P* < 0.001, see Fig. 2A). Furthermore, ADC_water_ showed a significant negative correlation with the relative concentration of NAA (*β* = −0.226, *P* < 0.01, Fig. 2B) and a significant positive correlation with the relative concentration of myo-inositol (*β* = 0.327, *P* < 0.001, Fig. 2C and Supplementary Table 2). When the relative concentration of both NAA and myo-inositol were included as predictors in the model, ADC_water_ was significantly associated only with the relative concentration of myo-inositol (NAA: *β* = −0.069, *P* > 0.1; mIns: *β* = 0.294, *P* < 0.001). Conversely, ADC_NAA_ was significantly associated only with the relative concentration of NAA (NAA: *β* = 0.038, *P* < 0.05; mIns: *β* = −0.025, *P* > 0.1).

## Discussion

The present work demonstrates the potential of combining multiple neuroimaging techniques to study the microstructural changes in Alzheimer’s disease in a compartment and cell-specific manner. The most salient DW-MRS finding is that in the CI group, the diffusion rate of NAA (ADC_NAA_) in the PCC, a region showing elevated tau level in the CI group, was significantly lower than in the CU group. NAA is present in high concentration (above 10 mM) almost exclusively in neurons.^31^ This finding therefore supports the hypothesis that the build-up of intraneuronal tau aggregates hinders the cytosolic diffusion within neurons. This study presents the first evidence that the cellular specificity of DW-MRS could be exploited for monitoring the effect of tau accumulation on neurons, paving the way to a potential use of DW-MRS for tracking the effect of disease-modifying therapies targeting tau in Alzheimer’s disease.

In contrast to ADC_NAA_, ADC_water_ in the same cortical region was ‘higher’ in the CI than in the CU group and ADC_NAA_ was negatively correlated with ADC_water_. Considering the ‘relative concentrations’ of neuronal and glial metabolites allowed a more comprehensive interpretation of the results. Both ADC_NAA_ and ADC_water_ were significantly associated with the relative concentration of NAA. ADC_water_, however, was significantly associated with the relative concentration of myo-inositol, even independently from the relative concentration of NAA. Myo-inositol is preferentially found in astrocytes,^16^ therefore our results indicate that the increase in ADC_water_ can be partially explained by pathological events that occur outside neurons, and in particular by differences in astrocytic activity. Changes in ADC_water_ have been shown to be associated with astrocyte physiology in the mouse brain, in particular with the function of aquaporin-4.^32^ Recent findings have also shown a positive association between water diffusion in cortical regions and the plasma level of the glial fibrillary acid protein (GFAP), a peripheral marker of astrocytic activity.^6^ Taking these together, our results suggest that different events, possibly tau accumulation in neurons and astrocytic activation, affect the diffusion of NAA and water differently.

Several limitations should be considered when interpreting the results of this study. The small sample size limits the generalizability of the results and further studies should replicate and expand the current analyses. Considering the exploratory nature of the study, we focused on the comparison between Aβ-negative cognitively unimpaired subjects and cognitively impaired patients with evidence of both amyloid and tau accumulation. We did not include, however, groups at intermediate stages of the Alzheimer’s disease continuum, e.g. participants with evidence of significant Aβ but not tau accumulation that should be studied in future works. Investigating this group of participants allows to study a potential dissociation between ADC_NAA_ and ADC_water_. We expect that ADC_NAA_ would not show a significant decrease in participants who are Aβ-negative but do not exhibit evidence of tau accumulation, while ADC_water_ could be higher in this group. Confirming the specificity of ADC_NAA_ to tau accumulation could open the way to the development of DW-MRS-based biomarker for monitoring the effect of anti-tau treatments that are currently under development and being tested in clinical trials. However, the validation of a new biomarker was beyond the scope of the present study and it would require measuring ADC_NAA_ also in regions that are affected by tau accumulation early in the disease process, like the medial temporal lobe. Another possible limitation to the clinical translation of the current results is related to the relative lower availability of 7 T MRI scanner when compared to 3 T MRI scanners. 7 T MRI provides higher sensitivity to spectroscopic data, but mitigating this caveat are results from a two-centre study in which the ADC_NAA_ obtained at 7 T was shown to be highly reproducible using a 3 T scanner, with similar variance across the two cohorts.^33^

With this limitation in mind, our study demonstrates that a multifactorial approach that considers diffusion of NAA and water, as well as the relative concentrations of neuronal and glial markers, can provide complementary information for a more comprehensive interpretation of cortical microstructural alterations occurring in Alzheimer’s disease. Such information could be harnessed to develop new sensitive markers to downstream effect of protein accumulation.^21^

## Supplementary Material

fcae026_Supplementary_Data

## Data Availability

Anonymized data will be shared by request from a qualified academic investigator for the sole purpose of replicating procedures and results presented in the article and as long as data transfer is compliant with EU legislation on general data protection regulations (GDPR) and decisions made by the Swedish Ethical Review Authority and Region Skåne.
