# Supplementary material for: Diffusion weighted magnetic resonance spectroscopy revealed neuronal specific microstructural alterations in Alzheimer’s disease
Source: Brain Commun. 2024 Feb 1;6(1):fcae026. doi: 10.1093/braincomms/fcae026 (PMC10873577; doi:10.1093/braincomms/fcae026)
Supplement: fcae026_Supplementary_Data [file fcae026_supplementary_data.docx]

**Supplementary materials**

**Materials and Methods**

**Inclusion and exclusion criteria for participants in the Swedish BioFINDER-2 study**

The Swedish BioFINDER-2 study enrolls participants in five sub-cohorts.

Cohort A and B include neurologically and cognitively healthy controls. The inclusion criteria are: i) ages 40-65 years (cohort A) and ages 66-100 years (cohort B); ii) absence of cognitive symptoms as assessed by a physician specialized in cognitive disorders; iii) Mini Mental State Examination (MMSE) score 27-30 (cohort A) or 26-30 (cohort B) at screening visit; iv) do not fulfill the criteria for MCI or any dementia according to DSM-5 ^1^; v) fluent in Swedish.

Cohort C comprises participants with subjective cognitive deficits (SCD), or mild cognitive impairment (MCI; defined as a performance of <-1.5 SD below reference mean in at least one cognitive domain, see ^2^ for further details). Inclusion criteria are: i) Age 40-100 years; ii) referred to memory clinic due to cognitive symptoms; iii) MMSE score of 24 – 30 points; iv) does not fulfill the criteria for any dementia (major neurocognitive disorder) according to DSM-5, v) fluent in Swedish.

Cohort D consists of participants with dementia due to AD. Inclusion criteria are: i) Age 40-100 years; ii) referred to memory clinic due to cognitive symptoms; iii) MMSE score of > 12 points; iv) fulfill the DSM-5 criteria for dementia (major neurocognitive disorder) due to Alzheimer’s disease^1^;v) fluent in Swedish.

Cohort E covers other non-AD dementias and neurodegenerative disorders. Exclusion criteria for all sub-cohorts are: i) significant unstable systemic illness that makes it difficult to participate in the study; ii) current significant alcohol or substance misuse; iii) refusing lumbar puncture, MRI or PET.

The participants in the present study have been enrolled from cohort A, B and C (only people with SCD) of the BioFINDER-2 study for the cognitively unimpaired group and from cohort C and D for the cognitively impaired group. All participants were assessed by physicians with expertise in dementia disorders.

**MRS-MRI 7T protocol**

7T MRI scans were performed on a Philips Achieva whole-body scanner (Philips Healthcare, The Netherlands) equipped with a quadrature transmit/32-channel receive head coil (Nova Medical, USA) and gradient coils with a maximum gradient strength of 40 mT/m and a slew rate of 200 T/m/s.

*Single-volume water-suppressed MRS data:* Water suppression was achieved using the variable pulse power and optimization relaxation delays (VAPOR) sequence^2^. Offset independent trapezoid (OIT) refocusing pulses were used to minimize in-plane chemical shift displacement errors. The basis-set for the MRS analysis included a total of 23 metabolites (including the MM baseline). The metabolites included are the following: alanine (Ala), ascorbate (Asc), aspartate (Asp), creatine (Cr), gamma-aminobutyrique (GABA), glucose (Glc), glutamine (Gln), glutamate (Glu), glycine (Gly), glycerophosphocholine (GPC), glutathione (GSH), myo-inositol (mIns), lactate (Lac), N-acetyl-aspartate (NAA), NAA-glutamate (NAAG), phosphocholine (PCho), phosphocreatine (PCr), phosphatidylethanolamine (PE), scyllo-inositol (sIns), serine (Ser), taurine (Tau), threonine (Thr), and the MM baseline.

*Single-volume DW-MRS data:* Data were collected along 3 orthogonal directions [1 1 –0.5], [-0.5 1 1], and [1 –0.5 1]), providing maximum combined gradient amplitudes. Overall, each diffusion condition was repeated 24 times for metabolites and 4 times for water acquisitions. The total NSA was 3 (encoding directions) x 2 (b-values) x 24 (NSA/condition) = 144 for metabolite data, and 24 for water acquisitions (4 NSA/condition). For metabolite acquisitions, water suppression was achieved using two frequency-selective excitation pulses, each followed by a dephasing gradient.

**Supplementary tables**

**Supplementary Table 1**: Quality control on ^1^H DW-MRS data (SNR, FWHM and CRLB) and ADC of metabolites.

|  |  | CU | | | CI | |
| --- | --- | --- | --- | --- | --- | --- |
| SNR | *b1, dir1*  *b1, dir2*  *b1, dir3*  *b2, dir1*  *b2, dir2*  *b2, dir3* | 12 ± 3  15 ± 3  14 ± 3  10 ± 2  10 ± 3  9 ± 2 | | | 10 ± 2  11 ± 2  12 ± 2  9 ± 2  8 ± 2  8 ± 2 | |
| FWHM (Hz) | *b1, dir1*  *b1, dir2*  *b1, dir3*  *b2, dir1*  *b2, dir2*  *b2, dir3* | 8 ± 1  9 ± 1  8± 1  9 ± 2  9 ± 1  11 ± 2 | | | 9 ± 1  9 ± 1  9 ± 1  10 ± 2  10 ± 1  10 ± 2 | |
|  |  | **CRLB (%)** | | **ADC (μm^2^/ms)** | **CRLB (%)** | **ADC (μm^2^/ms)** |
| NAA | *b1, dir1*  *b1, dir2*  *b1, dir3*  *b2, dir1*  *b2, dir2*  *b2, dir3* | 3 ± 1  2 ± 1  3 ± 1  3 ± 1  4 ± 1  4 ± 1 | 0.124 ± 0.011 | | 3 ± 1  3 ± 0  3 ± 0  4 ± 1  4 ± 1  4 ± 1 | 0.118 ± 0.008 |
| tCr | *b1, dir1*  *b1, dir2*  *b1, dir3*  *b2, dir1*  *b2, dir2*  *b2, dir3* | 4 ± 1  3 ± 1  3 ± 1  4 ± 1  4 ± 1  4 ± 1 | 0.120 ± 0.011 | | 4 ± 1  3 ± 1  3 ± 1  4 ± 1  4 ± 1  4 ± 1 | 0.113 ± 0.013 |
| tCho | *b1, dir1*  *b1, dir2*  *b1, dir3*  *b2, dir1*  *b2, dir2*  *b2, dir3* | 6 ± 2  5 ± 1  5 ± 1  6 ± 2  6 ± 2  7 ± 2 | 0.107 ± 0.017 | | 6 ± 1  5 ± 1  5 ± 1  6 ± 1  6 ± 2  7 ± 2 | 0.109 ± 0.013 |

The SNR and FWHM are output measures given by LCModel. SNR is defined as the ratio of the maximum in the spectrum-minus-Baseline over the analysis window to twice the residuals RMS. FWHM (given in Hz) corresponds to the approximate full-width at half maximum of the singlets in the Basis Set. The CRLB values are given for each metabolite.

**Supplementary Table 2**: Quality control on ^1^H MRS data (SNR, FWHM and CRLB) and concentrations of metabolites (normalized to tCr).

|  | CU | | CI | |
| --- | --- | --- | --- | --- |
| SNR  FWHM (Hz) | 34 ± 6  10 ± 2 | | 29 ± 4  10 ± 1 | |
|  | **/Cr** | **CRLB (%)** | **/Cr** | **CRLB (%)** |
| Ins | 0.72 ± 0.06 | 3.4 ± 0.7 | 0.89 ± 0.10 | 2.9 ± 0.6 |
| Glu | 1.10 ± 0.09 | 2.9 ± 0.5 | 1.08 ± 0.08 | 3.1 ± 0.6 |
| Gln | 0.33 ± 0.03 | 10.3 ± 1.8 | 0.37 ± 0.07 | 10.1 ± 2.3 |
| GSH | 0.17 ± 0.02 | 9.9 ± 2.1 | 0.17 ± 0.03 | 10.8 ± 2.8 |
| Asp | 0.37 ± 0.07 | 13.9 ± 3.9 | 0.35 ± 0.06 | 15.9 ± 3.7 |
| Tau | 0.22 ± 0.04 | 10.8 ± 3.9 | 0.24 ± 0.05 | 10.6 ± 2.3 |
| Scyllo | 0.04 ± 0.02 | 20.2 ± 17.1 | 0.03 ± 0.02 | 36.6 ± 31.0 |
| MM | 0.17 ± 0.02 | 3.0 ± 0.6 | 0.16 ± 0.02 | 2.9 ± 0.4 |
| NAA | 1.29 ± 0.08 | 1.9 ± 0.3 | 1.17 ± 0.11 | 1.9 ± 0.3 |
| tCr | - | 1.9 ± 0.3 | - | 2.0 ± 0.0 |
| tCho | 0.19 ± 0.02 | 4.2 ± 1.3 | 0.22 ± 0.03 | 3.6 ± 1.4 |

The SNR and FWHM are output measures given by LCModel. SNR is defined as the ratio of the maximum in the spectrum-minus-Baseline over the analysis window to twice the residuals RMS. FWHM (given in Hz) corresponds to the approximate full-width at half maximum of the singlets in the Basis Set. The CRLB values are given for each metabolite.

**Supplementary figures**

Supplementary Figure 1: **(A)** **Illustration of MRI data processing pipeline.** 7T 3D T1w images were segmented using FSL to generate GM mask. SUV and ADC_water_ maps were then registered to the 7T 3D T1w images and the GM mask was applied to extract GM-SUV and GM-ADC_water_ maps. Finally, the volume-of-interest (VOI) used for MRS measurement was used as a mask to extract GM fraction, GM-SUV and GM-ADC_water_ from the VOI. **(B)** **Illustration of the MRS data processing pipeline**, showing the metabolites’ fit obtained using LCModel for one cognitively impaired subject. **(C) Illustration of the DW-MRS data** obtained at two b-values along one of the three directions for one cognitively impaired subject.


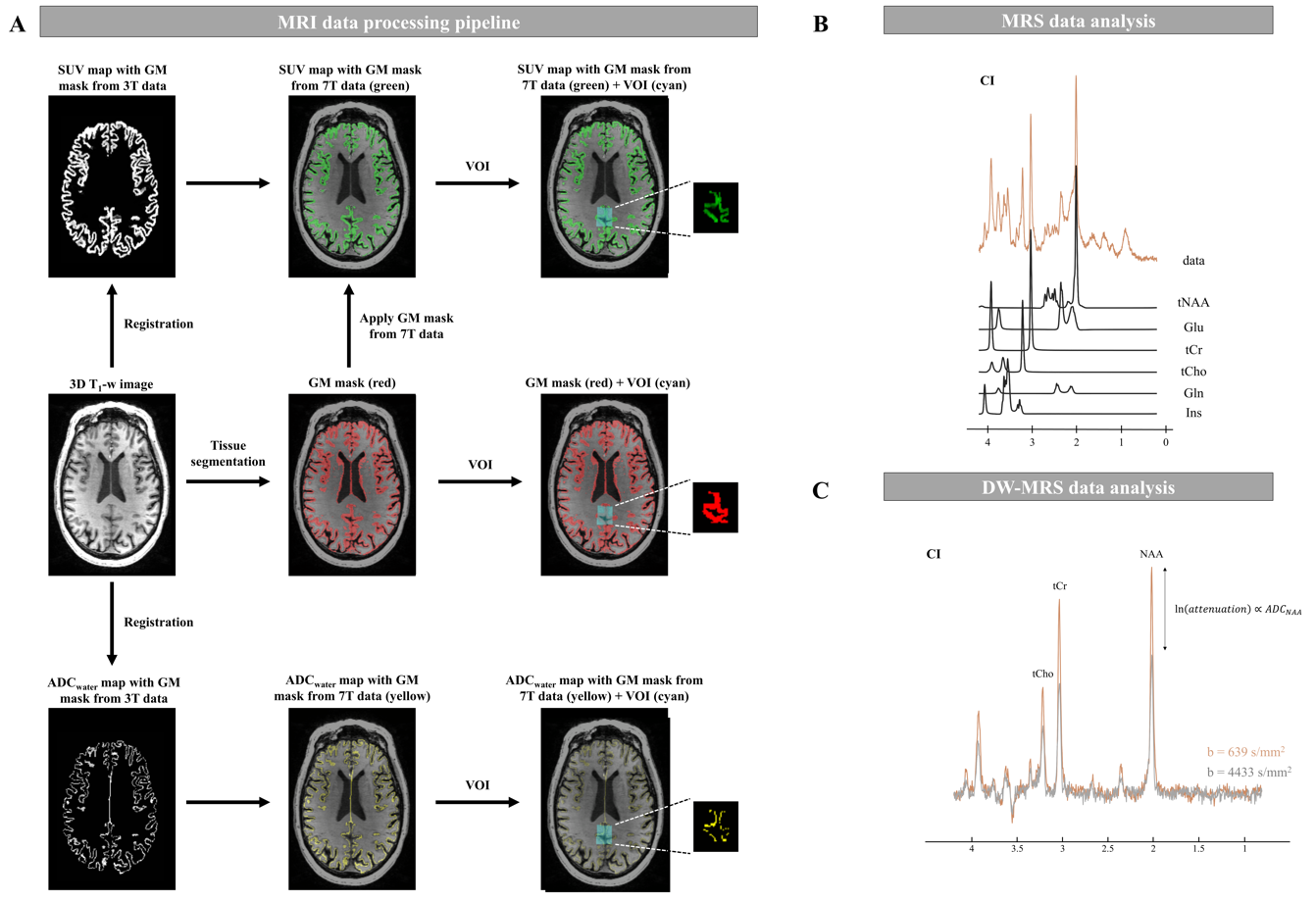


**Supplementary references**

1. American Psychiatric Association. *Diagnostic and Statistical Manual of Mental Disorders*. American Psychiatric Association; 2013. doi:10.1176/appi.books.9780890425596

2. Tkác I, Starcuk Z, Choi IY, Gruetter R. In vivo1H NMR spectroscopy of rat brain at 1 ms echo time. *Magn Reson Med*. 1999;41(4):649-656. doi:10.1002/(SICI)1522-2594(199904)41:4<649::AID-MRM2>3.0.CO;2-G
